# Does chief executive compensation predict financial performance or inaccurate financial reporting in listed companies: A systematic review

**DOI:** 10.1002/cl2.1370

**Published:** 2023-12-11

**Authors:** Denise Rousseau, Byeong Jo Kim, Ryan Splenda, Sarah Young, Jangbum Lee, Donna Beck

**Affiliations:** ^1^ Heinz College and Tepper School of Business Pittsburgh Pennsylvania USA; ^2^ Graduate School of Public Administration Seoul National University Seoul South Korea; ^3^ University Libraries Carnegie Mellon University Pittsburgh Pennsylvania USA; ^4^ University Libraries Pittsburgh Pennsylvania USA

## Abstract

**Background:**

Financial incentives for chief executive officers (CEOs) are thought to motivate them to lead their company toward achieving important business objectives. Based on the Rousseau et al. (2019) protocol, this systematic review assesses the predictive effects of CEO incentives on certain business outcomes.

**Objectives:**

This review addresses whether CEO financial incentives predict: (1) firm financial performance and (2) financial restatement due to misreporting.

**Search methods:**

We searched nine research databases for published peer‐reviewed literature (to July 23–26, 2021 and an attenuated search from those dates to July 27–31, 2023) and thirteen professional association websites for non‐published gray literature (to August 2021). We also hand‐searched selected relevant journals.

**Selection criteria:**

We reviewed peer‐reviewed and unpublished studies available in English since 1980. Eligible studies regarding our first question assessed CEO financial incentives (1) 1 year or more before the measurement of outcomes, (2) controlled for pre‐incentive firm performance or market conditions, and (3) analyzed CEO financial incentives as predictors of firm outcomes. Eligible studies regarding our second question assessed whether financial restatement had occurred and analyzed effects of CEO incentives on this outcome.

**Data collection and analysis:**

We extracted standardized regression coefficients for each effect or converted unstandardized regressions to standardized. Analyses were conducted using STATA. All studies were assessed to have moderate risk of bias.

**Main results:**

For our first question, 20 studies (15,398 firms) met our criteria for meta‐analysis of effects. Bonuses, the most commonly studied incentive, had a small positive effect on next year's accounting performance metric Return on Assets (ROA, 0.046 [*k* = 7, 95% confidence interval (CI) = 0.014, 0.078]). The bonus effect in the market‐related metric of Stock Returns (−0.026 [*k* = 5, 95% CI = −0.119, 0.067]) fell within a CI including 0, as did its effect on another market‐related metric, Market‐to‐Book value (Tobin's Q, 0.028 [*k* = 3, 95% CI = −0.024, 0.08]). We conclude that Bonuses show no predictive effect on the following year's market‐related metrics but do affect ROA. Stock Options had no effect on next year's ROA (0.027 [*k* = 5, 0.95% CI = 0.000, 0.052]), nor on Market‐to‐Book Value (Tobin's Q, 0.097 [*k* = 5, 95% CI = −0.027, 0.220]) or Stock Return (0.042 [*k* = 6, −0.033, 0.117]), indicating no predictive effect for Stock Options on either accounting or market‐related performance. We sought but found too few studies to report on effects of incentives on other financial outcomes or for lags greater than 1 year. For our second question, three studies (*n* = 2044 firms) met our criteria. The overall effect size for CEO Incentives on Restatement (−0.09 [*k* = 3, 95% CI = −0.363, 0.184) fell within a CI including zero. We conclude that current evidence does not support a direct relationship between CEO financial incentives and Restatement.

**Authors' conclusions:**

This review affirms a small effect of CEO Bonuses, but no effect of Stock Options, on the accounting performance metric ROA. In contrast, neither Bonuses nor Stock Options predict a firm's market‐related metrics. CEO incentives also are unrelated to Financial Restatement. Despite widespread use of CEO financial incentives, lack of evidence supporting their use, beyond the bonus‐ROA effect we identify, suggests caution regarding current CEO financial incentive practice and greater consideration of alternative arrangements to enhance firm performance.

## 1 PLAIN LANGUAGE SUMMARY

### 1.1 Bonuses for Chief Executive Officers (CEOs) predict next year's return on assets (ROA), but stock options do not

CEO bonuses have a small predictive effect on next year's ROA but do not affect other performance metrics. CEO stock options show no effect on firm performance metrics.

This systematic review also finds that CEO financial incentives are unrelated to subsequent financial restatements, which are changes in reports of business outcomes due to inaccuracies or errors.

### 1.2 What is this review about?

CEO financial incentives are intended to motivate a firm's CEO to attain important business goals. CEO pay in the USA has risen nearly a thousand fold since 1978, relative to a 12% increase for rank and file employees.

The financial incentives covered in this review include the most frequently‐studied incentives: bonuses for achieving business targets and stock options on favorable terms, increasingly a major source of CEO wealth.

In addition, this review examines the effect of CEO financial incentives on subsequent financial restatement of firm results. Since the company's employees compile the data used to assess their firm's performance, it creates the potential for bias and thus concern regarding the effect of CEO incentives on financial misreporting and subsequent financial restatement.

No systematic review has been conducted on the effects of financial incentives to CEOs, so firm compensation committees and policymakers have had no available synthesis of the empirical evidence to inform their decisions.

**What is the aim of this review?**

This Campbell systematic review examines the effects of CEO financial incentives on firm accounting, market performance and financial restatement.

### 1.3 What studies are included?

This review summarizes the statistical effects of 20 empirical studies over 40 years that investigated the extent to which financial incentives predict subsequent firm performance, typically for the following year. It also summarizes the results of three studies on the relationship between CEO financial incentives and subsequent financial restatement of business outcomes.

We include studies conducted between 1980 and 2023: the era of deregulation and increased competition begun under the administrations of Reagan in the USA and Thatcher in the UK.

Included studies are from publicly traded firms in which CEO financial incentives at one point in time were obtained retrospectively to investigate their predictive effects on subsequent outcomes.

Studies represent firms across the globe but mostly carried out in the USA, Europe and Australia.

Studies all had potential methodological weaknesses and typically failed to report their treatment of missing data, rationale for controls used, or the set of analyses conducted before reporting final results. None used experimental designs. Included studies are those reported in English.

### 1.4 What are the main findings of this review?

CEO bonuses have a small positive predictive effect on next year's ROA but have no effect on either next year's market‐to‐book value (Tobin's Q) or stock return.

Stock options have no effect on next year's ROA or any market‐related metrics.

Too few studies exist to test other time lags or incentive effects on other outcomes. CEO financial incentives have no effect on financial restatement.

CEO financial incentives are unrelated to subsequent financial restatement.

### 1.5 How up‐to‐date is this review?

The review authors searched for studies up to 2021 and then updated it through 2023 by searching the two most productive research databases.

## 2 BACKGROUND

### 2.1 Description of the condition

CEO incentive compensation is intended to motivate chief executives to help their firms attain important business goals by aligning CEO interests with those of the firm's stakeholders, including investors, employees, and others. Targets may be short‐term such as annual increase in stock price, or long‐term such as revenue growth over a period of years. Such financial performance metrics are typical performance targets used in CEO incentive compensation. They provide evidence of the firm's financial well‐being and future prognosis, both of interest to the firm's current stockholders and future investors. However, business outcomes compiled by a firm's employees are potentially subject to inaccurate reporting. This inaccurate reporting is attributable at times to errors and other times to bias in the information reported as a result of direct and indirect influence by the CEO and that person's direct reports, which can result in fraud. Financial restatements are changes in reports of business outcomes due to inaccuracies or errors identified through audits by company accountants or outside auditors.

### 2.2 Description of the intervention

Incentive compensation refers to formal contracts to provide a bonus or compensation increase contingent on the firm attaining a performance target or targets—a common feature of publicly traded firms. A recent report by the compensation research firm Equilar compiled data reflecting pay for the chief executives at 199 public companies, indicating that over 95% had incentive compensation (the remaining handful of firms had CEOs with substantial ownership in the firm) (“Pay at the Top,” 2011). Incentive compensation for meeting performance targets like total revenue, change in net income, or change in shareholder return can include cash bonuses (Ashley & Yang, 2004; Coleman, 2000; Nourayi & Mintz, 2008) as well as equity compensation (e.g., stock options, restricted stock) (e.g., Ashley & Yang, 2004; Jeppson et al., 2009).

### 2.3 How the intervention might work

Incentives are expected to direct CEO attention to certain outcomes and away from others. Moreover, since CEOs can negotiate their own contracts, they may bargain for terms they believe are more achievable. Incentives also can motivate CEOs to direct the activities of their subordinates to achieve the financial performance outcomes specified in their contracts. However, this attention to specific outcomes can lead to inaccurate financial reporting if the CEO or subordinates manipulate the firm's financial data to allow the CEO to receive the incentives contracted. Such manipulation often results in the need to correct or restate previously filed financial information. The regulatory environment may influence the incidence of financial restatements and their link to financial incentives: Research suggests that the efforts at deterrence and detection of financial misstatements by public companies have been successful, with only a small portion of restatements by public companies in recent years considered fradulent after the Sarbanes‐Oxley Act of 2002 made various changes in financial reporting requirements (cf. Alali & Wang, 2017).

### 2.4 Why it is important to do this review

This review examines CEO incentive compensation, a major organizational decision with implications for stakeholders, firms, and society. It comes at a time when CEO pay in the US has risen 940% since 1978 relative to a 12% increase for rank and file employees (Mishel & Wolfe, 2019). For governing boards of corporations, CEO pay is a major decision with potential implications for firm performance, effective use of resources, employee well‐being, and long‐term organizational consequences. Since CEOs negotiate their own contracts, they may bargain for terms more favorable to themselves than to the firm's other stakeholders. Moreover, incentives can have unintended consequences including manipulating accounting data to increase the likelihood of receiving contracted incentives. Disputes regarding the efficacy of CEO incentive compensation include whether CEOs are paid too much relative to the salaries of rank and file employees or their contribution to the firm's success.

A multidisciplinary review (Devers et al., 2007) suggests that executive compensation (i.e., of top management generally and not only the CEO) is a reward for prior performance and need not necessarily contribute to subsequent firm performance. Tosi et al. (2000) conducted a meta‐analysis of determinants of CEO pay, finding that 40% of the variance in total CEO pay is attributable to firm size, while (past) firm performance accounts for less than 5%. In their widely cited critique of CEO incentives, Jensen and Murphy (1990) maintain that CEOs should be paid based on increases in corporate value as measured by their contribution to shareholder gains, advocating CEOs should hold substantial amounts of company stock. We note, however, that limited research exists in support of their claim.

## 3 OBJECTIVES

One goal of this systematic review is to assess whether incentive terms in CEO contracts predict subsequent firm financial performance; a second goal is to identify whether incentive terms in CEO contracts are related to inaccurate financial reporting as manifest in restatement of accounting data due to errors or other distortions in reporting those data.

## 4 METHODS

### 4.1 Criteria for considering studies for this review

#### 4.1.1 Types of studies

The protocol for this review was published in 2019 (Rousseau et al., 2019).

Because we focus on the prediction of firm performance and financial restatements by CEO financial incentives, this review requires evidence that predictors (incentives) occur before outcomes. Eligible studies are longitudinal in nature with financial outcomes and/or final restatement measured at later point in time than the incentive measures. Included studies used controls for (a) pre‐incentive firm performance and/or (b) market conditions prevailing at the time longitudinal firm performance measures are gathered (e.g., random effects [luck] that can increase market‐related outcomes, such as increase in oil price for firms in the petroleum industry [Bertrand & Mullainathan, 2001]). Eligible studies include those where CEO financial incentives served as predictors for subsequent financial outcomes and could include comparison groups where incentives differ. Only restatement studies used comparison groups. In studies with financial performance outcomes, studies tested the direct effects of financial incentives controlling for past performance across firms using a variety of additional controls including firm size and CEO attributes (e.g., age or tenure).

#### 4.1.2 Types of participants

Included studies are limited to those that focus on publicly traded firms. Studies that focus on private companies were excluded. Included studies were limited to those examining the incentive contracts of CEOs. (We excluded studies that focused on non‐CEO executive positions such as Chief Operating Officers or a set of top executives rather than CEOs alone.)

#### 4.1.3 Types of interventions

Incentive contracts in a publicly traded company include all formal agreements entered into between a corporate board and the CEO in which future rewards are offered contingent on attaining specified levels of performance or performance targets. Such contracts can include cash bonuses, stock options and other financial instruments offered based on the attainment of future performance outcomes. (Note incentives are distinct from pre‐specified salary or pension levels but can include salary increases commited in advance for attainment of performance targets.)

N.B. Randomized controlled studies are unlikely in this context and none were found in our search.

#### 4.1.4 Types of outcome measures

##### Primary outcomes

Our first primary outcome is firm financial performance. We identified the financial outcomes studies report, categorizing them according to their time frame (1 year, 2–3 years, 4+ years) and type of performance, that is, *profitability indicators* including Return on Investment (ROI), ROA, Return on Equity (ROE), Return on invested Capital Assets (ROIC), Return on Capital (ROC), Return on Sales (ROS), and Earnings before Taxes, Interest, Depreciation, and Amortizations (EBITDA) and *market returns* including changes in market‐to‐book value and other indicators of increased shareholder returns. We thus recorded the time lag reflected in each financial outcome and analyzed outcome data as a function of their time lag, that is, for example, grouping ROI measures at Year 1 together, then ROI measures at Year 2, and so forth. However, our statistical analyses focus on 1 year lags, as too few studies with longer lags are represented for a given incentive/outcome combination.

Our second primary outcome is whether financial restatements have been made. Financial restatements are corrections to previously issued accounting results for a firm and are used as an indicator of manipulation or misspecification of outcomes attained during a CEO's tenure.

All outcomes were derived from archival data as reported in studies included in this review. We include both studies with useable data and those whose data are ultimately deemed unuseable for constructing effect sizes in order capture what is known in relevant studies regarding the effects addressed in this review. For studies with incomplete information that otherwise fit our inclusion criteria, we attempted to obtain information from authors to increase the useability of their data.

##### Secondary outcomes

None.

### 4.2 Search methods for identification of studies

The search strategy for this review was developed by information specialists (R. S. and S. Y.) with the aim of identifying research focused on CEO incentive compensation and measuring publicly traded firms' financial performance and/or financial restatements. Studies reported in English since January 1, 1980 were included. This time frame was used to target findings in the economic era that began with Margaret Thatcher becoming Prime Minister in the UK in 1979 and Ronald Reagan becoming President of the United States in 1981, and the resulting changes in tax structure and corporate regulation and de‐regulation initiated and sustained since then.

#### 4.2.1 Electronic searches

We conducted a systematic search of all electronic databases listed in Table 1 in December 2019. An update search of the same databases was performed between July 23–26, 2021 (and later updated this search to July 2023, see below). The title/abstracts were screened by two reviewers as was the full text of the potentially eligible studies. The platforms and databases listed were selected because they provide both disciplinary and interdisciplinary research coverage of our research questions. We made no language restriction in our initial search to investigate the incidence of potentially relevant primary studies in languages other than English. Less than a dozen studies in other languages were identified, sometimes accompanied by an English language summary. In the latter case, no study met our criteria for inclusion so we did not seek further translation.

**Table 1:** Electronic search platforms and databases.

Database	Platform
ABI/INFORM	ProQuest
Business Source Ultimate	EBSCO
Emerald Insight	Emerald
EconLit	EBSCO
Directory of Open Access Journals	https://doaj.org/
Dissertations and Theses Global	ProQuest
Scopus	Elsevier
Web of Science Core Collection (see Supporting Information: File for list of sub‐databases)	Clarivate

Titles, abstracts, and other citation information for all search records (both the original 2019 search and the 2021 and 2023 updates) in English were exported as RIS files and uploaded into Zotero for de‐duplication. Once de‐duplication was completed, all records were then uploaded into the Covidence platform for Title and Abstract screening and full text review.

Subsequent to the first two full searches, we sought further updating to 2023 by creating a search summary table, which facilitates the analysis of the source contributions to the final included studies (Bethel et al., 2021). Based on this analysis, we chose the two databases collectively contributing the most studies to the included studies (Scopus, which contributed 11 and EconLit which contributed an additional 3). We reran searches limiting the date range to 2021–2023 in these two databases on July 27 and July 31, respectively, yielding a total of 767 new records.

#### 4.2.2 Searching other resources

##### Gray literature

In addition to the bibliographic databases mentioned above, we searched a number of gray literature resources, including conference proceedings, white papers, working papers, and other types of information from January 1, 1980 to present that are listed in Table 2. For resources that allow for advanced searching, we developed search strategies and report on those in Supporting Information: File 1. Both advanced and basic keyword searches for these resources focused on terms related to CEO compensation, firm financial performance, and financial restatements. Any gray literature reported before January 1, 1980 was excluded and date limits were applied to these searches when available. In most cases, results were screened within each gray literature platform and only relevant results were retrieved and uploaded into Covidence for Title and Abstract screening and full text review. Initial searching of these resources took place in February 2020, and a search update was conducted in August 2021 and July 2023.

**Table 2:** Gray literature and conference proceeding searching.

Organization/conference	Website
Academy of Internatioanl Business (AIB) Proceedings	https://aib.msu.edu/publications/confproceed.asp
American Economic Association (AEA) Papers & Proceedings	https://www.aeaweb.org/journals/pandp
Board of Governors of the Federal Reserve System	https://www.federalreserve.gov/publications.htm
Bureau of Economic Analysis (BEA)	https://www.bea.gov
Business Council of Canada	https://thebusinesscouncil.ca
Center for Economic Policy Research (CEPR)	https://cepr.org
Conference Board ‐ Business Management Research	https://www.conference-board.org/ea/search.cfm
Federal Reserve Economic Data (FRED)	https://fred.stlouisfed.org
International Conference on Advances in Management Sciences (ICAMS) ‐ Journal of Advanced Management Science	http://www.joams.com/index.php?m=content&c=index&a=lists&catid=9
International Conference on Economics, Business and Management (ICEBM) ‐ Journal of Economics, Business and Management	http://www.joebm.com/list-6-1.html
National Bureau of Economic Research (NBER) Working Papers	https://www.nber.org/papers.html
Research Papers in Economics (RePEc) IDEAS	https://ideas.repec.org
Social Sciences Research Network (SSRN)	https://www.ssrn.com/en

##### Hand searching

To help supplement both electronic and gray literature searching, hand searching was performed in select journals. We screened tables of contents and reference sections in Advances in Business Research (https://journals.sfu.ca/abr/index.php/abr), Academy of Management Review (https://journals.aom.org/journal/amr), and Academy of Management Annals (https://journals.aom.org/journal/annals).

Advances in Business Research, an open access journal hosted by San Francisco University, was searched from its inception in 2010 to the present day because it is not indexed in any of the above‐mentioned bibliographic databases. The references published in the Academy of Management Review and Academy of Management Annals were searched for the most current 1‐year timeframe, and also included “In‐press” articles. More information can be found in Supporting Information: File 1.

##### Supplementary searching

Once the final list of included studies was established, we performed both forward and backward citation searching to identify any additional relevant studies. Our forward searching method involved searching Google Scholar for any citing references of included studies. If there were zero citing references, a check within both the Web of Science and Scopus databases was done for completeness. Our forward searching method yielded an additional 92 relevant records to be screened. For backward searching, we hand‐searched the reference section of all included studies and identified an additional 35 relevant records for screening.

A number of leading experts in the field of compensation and contracts research were contacted by the lead author (D. R.) in Fall 2019. The experts were given the objectives of the systematic review and the types of studies desired for analysis. Most experts identified a handful of relevant studies that were saved for future reference. All of the relevant studies that experts identified were captured within the search strategies that were developed for bibliographic databases. The reference sections of related literature reviews published after January of 1980 were reviewed for any additional studies of interest. All potentially relevant studies were also captured in the abovementioned search strategies.

### 4.3 Data collection and analysis

#### 4.3.1 Selection of studies

All results from database and gray literature searches were added to Covidence, to manage the process of deduplication and study screening. After all searches were conducted and the deduplication process was complete, two of the review authors independently screened all titles and abstracts, excluding studies that are clearly irrelevant to the review question. Any studies deemed to possibly meet inclusion criteria by at least one reviewer, or for which there is insufficient information to determine eligibility, were retrieved in full text. Two authors independently reviewed the full text of these studies to determine eligibility based on the criteria outlined in Supporting Information: Appendix. Any disagreements between reviewers were resolved by discussion and consensus. Studies excluded at this stage were assigned a reason for exclusion.

The eligibility criteria were piloted by the reviewers on a total of 10 studies and clarifications made to ensure that the criteria were correctly interpreted and applied by all reviewers.

**Figure 1:**
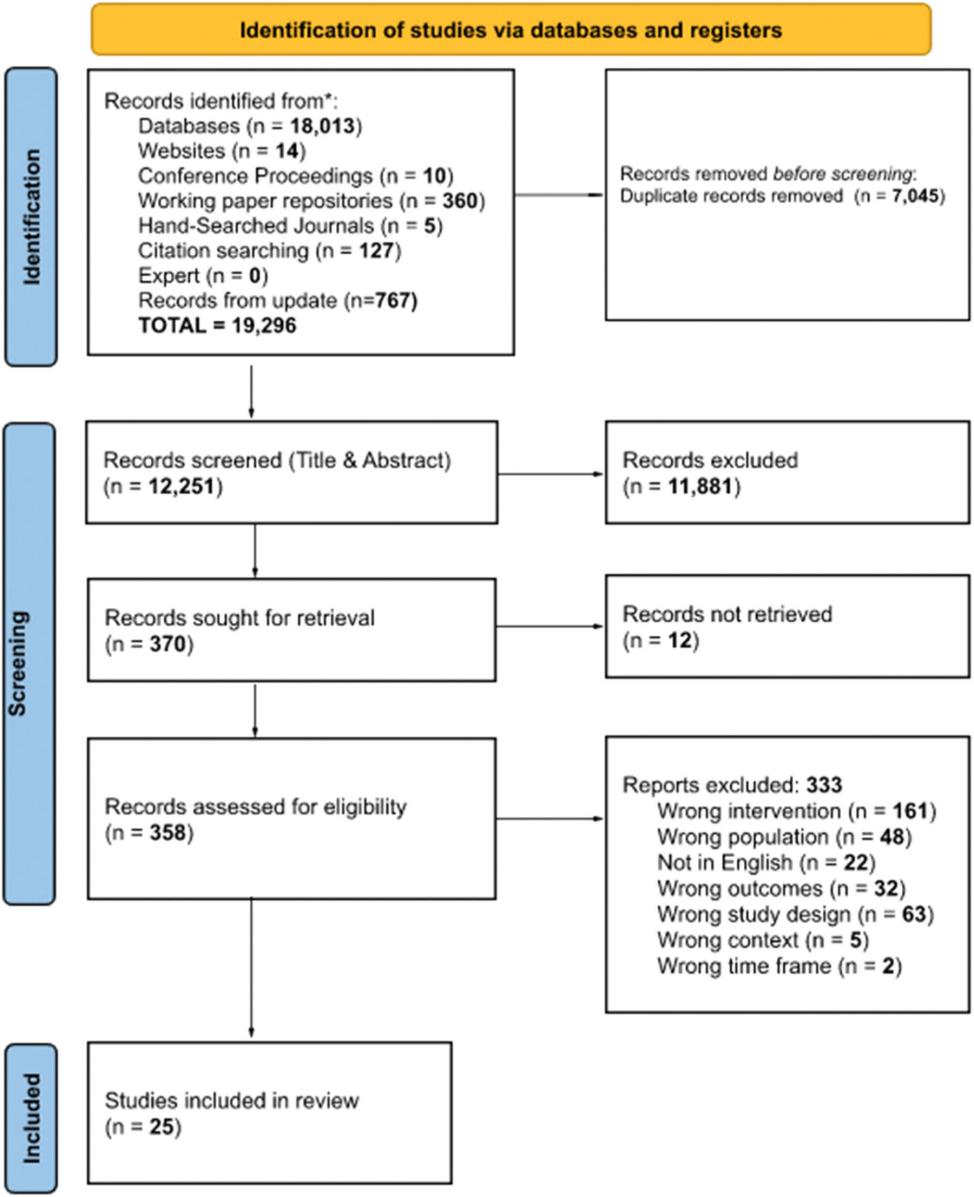
Prisma flow diagram.

Figure 1 provides a detailed breakdown of how the team arrived at the 23 studies included in our meta‐analyses. Note that additional studies related to our questions inform our systematic review although their data reporting was insufficient for meta‐analytic summary.

#### 4.3.2 Data extraction and management

Two review authors independently coded and extracted desired data from each of the included studies using the data extraction form in Supporting Information: Appendix. The data extraction form was piloted on a small number of studies and revised accordingly. Disagreements between reviewers were resolved through discussion and consensus. After this initial discussion, inter‐rater agreement regarding extracted data was high.

Where important information was missing in the case of three studies considered for extraction, we contacted the authors via email. In one case, the contacted author lacked the critical information (regarding time lags), attributing this to the passage of time since the study was conducted. In another, the contacted author indicated the executives studied were not CEOs. In a third case, a study published by a Chinese author team, repeated emails received no response. Though those emails were not returned, we do not know if authors actually received our requests for information.

#### 4.3.3 Assessment of risk of bias in included studies

Two raters assessed study‐level risk of bias after primary studies were identified and full‐text had been extracted. Risk of bias was assessed in terms of sample representativeness (of the population) and treatment of missing data. The clarity and reliability of performance measure reporting was evaluated. Raters also evaluated the appropriateness of controls used in analyses and whether alternative types of analyses were conducted or discussed to evaluate the possibility of p‐hacking (Wicherts et al., 2016). After initial coding of primary studies, the raters discussed differences to better specify criteria used in assessing bias. Subsequently raters agreed on their assessments.

#### 4.3.4 Measures of treatment effect

We use “r” as our indicator of treatment effect since our primary studies typically report findings as regression coefficients computed on data that are continuous and observational. Our indicator of a treatment effect is a “partial r” since we extracted regression coefficients controlling for past performance and typically other firm and CEO‐related factors. Our analyses used standardized coefficients; where only unstandardized were reported, we transformed them to standardized coefficients per advice from our statistical consultant David Choi. Our treatment effect controls for past firm performance, that is, levels of firm performance prior to or at the time the CEO's financial incentive contract is created. This control allows us to assess the degree of change in subsequent firm performance predicted by CEO financial incentives. Where reported effects included additional controls, we note these in our extraction tables.

#### 4.3.5 Unit of analysis issues

We focused on organization‐level studies to assess CEO effects in terms of organization‐wide outcomes as specified in CEO contracts. In studies where dependent effects existed, we followed Cochrane Handbook protocols by separating out analyses of specific effects by type of outcome (e.g., ROI, Stock Return) or time lag (1 year, 2 years, etc.). Since firms have only one CEO, included studies did not need adjustment for clustered data.

#### 4.3.6 Dealing with missing data

Incomplete information about studies was sought by contacting authors and searching for additional reports of those studies. We contacted three sets of authors of primary studies where it was unclear whether their study met our criteria regarding the focus on CEOs, controls for past performance, or effects of incentives on subsequent firm performance. One study was excluded when we were informed that it reported on incentives for a set of senior executives. Another study's senior author reported he no longer remembered the time frames involved. We received no reply from the third set of authors and omitted their study from consideration.

#### 4.3.7 Assessment of heterogeneity

Some substantive differences can exist including time periods studied (1980s to 2010s), firm size and country context. Methodological differences including analytic methods (regression vs bivariate analyses), differences in covariates and other factors related to risk of bias may also be factors and were coded to assess effects of heterogeneity. We used the Chi‐square test for heterogeneity and the tau‐squared statistic. We also examined forest plots to see if CIs for studies' effect sizes overlap.

#### 4.3.8 Assessment of reporting biases

We investigated the role of publication bias using meta‐regression on effect sizes using unpublished status as the reference.

#### 4.3.9 Data synthesis

We used STATA to conduct a random‐effects meta‐analysis, using the “partial r” metric for our effect size indicator. Random‐effect models assume underlying effects follow a normal distribution. They are used instead of fixed‐effect models that assume that all studies reflect the same population, attributing any differences to sampling error. We used inverse variance methods to weight study effect sizes by their precision in our meta‐analysis. We also used STATA to test for moderator effects (i.e., whether the relationship between incentives and outcomes depend on a third factor including publication bias or date of primary study publication).

#### 4.3.10 Subgroup analysis and investigation of heterogeneity

We intended to examine whether Risk of Bias levels affect effect sizes along with potential effects attributable to time frame and other factors. The total number of studies allowed us only to test for the general effects of publication bias and time frame of publication. Risk of bias was assessed as moderate across all studies using financial performance as the dependent variable and thus was not evaluated for differences in observed effects.

Metareg in STATA was used to test moderator effects.

#### 4.3.11 Sensitivity analysis

Sensitivity analysis was planned to address any deviations from the protocol made as a result of our review and analysis of the literature (e.g., changes in inclusion criteria). However, no deviations occured in our inclusion criteria and thus no sensitivity analyses are reported.

#### 4.3.12 Summary of findings and assessment of the certainty of the evidence

The most commonly studied CEO financial incentive, Bonuses, predicts only one subsequent firm financial oucome, specifically the accounting metric next year's ROA. The second most commonly studied CEO incentive, Stock Options, does not predict next year's ROA. Neither Bonuses nor Stock Options predict the market‐related metrics Stock Return or Market‐to Book value (Tobin's Q). Moreover, we find a publication bias in favor of significant results along with a moderate risk of bias in the primary studies included in our review due to lax reporting practices regarding missing data, choice of controls, and the set of analyses performed before actual reporting of findings (i.e., p‐hacking). With respect to Restatement, only three studies met our inclusion criteria, and the overall result indicates no effect of CEO incentives on Restatement. Given risk of bias, the limited number of relevant studies, and the tendency for publication bias, we infer that moderate uncertainty exists regarding the predictive effects of CEO financial incentives.

## 5 RESULTS

### 5.1 Description of studies

#### 5.1.1 Results of the search

The databases searches yielded 18,013 records, with an additional 516 records identified by Supporting Information: methods and gray literature searching. A total of 12,251 records were screened after deduplication across sources. Figure 1 shows the number of records retrieved and screened at each stage of the review. Twenty‐five studies met our general eligibility criteria and are included in the review with 23 reporting data amenable for inclusion in our meta‐analyses. (The two studies meeting our general criteria but not meta‐analyzed are Armstrong et al. [2010] due to a design using propensity matching and Webinger [2011] due to incomplete statistical information.)

#### 5.1.2 Included studies

Table 3 describes the included studies and Table 4 their summary statistics.

**Table 3:** Descriptive overview of included articles.

Article (in alphabetical order)	# of effect sizes	# Org	Tot. time	Lag	Publish	Type of incentive	Type of performance
Alangar (1993)	2	194	3‐year	1	NO	Bonus, Options	Performance
Al‐Shammari (2021)	2	204	1‐year	1	YES	Option pay	Tobin's Q, ROS
Balafas et al. (2014)	2	1787	13‐year	1	YES	Bonus	ROA, ROCE
Benson et al. (2019)	1	1500	17‐year	1	YES	CEO delta, CEO vega	Tobin's Q
Bradley (2013)	3	39	5‐year	1	YES	Bonus	ROA, ROE, EPS
Burns and Kedia (2006)	1	1500	11‐year	1	YES	Option sensitivity, Long‐term incentive payouts, sensitivity of equity, salary‐bonus sensitivity	Misreporting
Campbell and Weese (2016)	3	1500	4‐year	1	YES	Bonus, Stock grant, Stock options, pension	Tobin's Q
Chan et al. (2014)	6	68	13‐year	1, 3 & 5	YES	Bonus, Stock options	ROA
Covas (2004)	1	1822	10‐year	1	NO	Salary and bonus, Stock, Options	ROA
Gong et al. (2013)	1	2009	15‐year	1	YES	Incentive	Earning
Harris and Bromily (2005)	2	435	6‐year	1	YES	Bonus, Options	Misreporting
Hou (2012)	2	299	7‐year	1	NO	Long‐term performance based pay, Short‐term performance based pay	Stock return
Huang et al. (2010)	2	70	6‐year	1	YES	Bonus, Stock options	ROE, Stock return
Jin et al. (2017)	4	828	7‐year	1	YES	Percentage of average total bonus	Tobin's Q, ROA
Li et al. (2011)	1	64	13‐year	1	YES	Ratio of stock‐based compensation	ROA
Li et al. (2019)	3	106	25‐year	1	YES	equity‐based compensation	ROA, Tobins'Q, Stock return
Melinsky (2013)	1	109	5‐year	0 lag	NO	Bonus ratio	Restatement
Morrissey (2002)	1	560	8‐year	1	NO	Stock options	nibex (net income before extraordinary items and discontinued operations)
Noguera (2007)	4	113	5‐year	1 & 3	NO	Incentive‐based comp	ROA, Stock return
Smirnova et al. (2017)	2	330	4‐year	1	YES	Bonus	ROA, ROE
Smith and Swan (2008)	2	1500	10‐year	1	NO	Bonus	ROA, Tobin's Q
Stammerjohan (2004)	6	56	16‐year	1, 3 & 5	YES	Bonus‐percentageStock options percentage	Stock return
Weber (2006)	1	2349	21‐year	1	NO	Stock option	Stock return

**Table 4:** Descriptive statistics.

Article (in alphabetical order)	Sample size	CEO mean tenure/age		Others (industry and other qualitative characteristics)
Alangar (1993)	543		United States	
Al‐Shammari (2021)	204	6.52/55.29	United States	Manufacturing
Balafas et al. (2014)	6418	4.25/‐	United States	
Benson et al. (2019)	21,308	7.2/‐	United States	
Bradley (2013)	189	‐	South Africa	‐
Burns and Kedia (2006)	8208	6.80/‐	United States	
Campbell and Weese (2016)	4701	8.8/‐	United States	
Chan et al. (2014)	715		United States	Banking
Covas (2004)	8864		United States	
Gong et al. (2013)	716		United States	
Harris and Bromily (2005)	844		United States	
Hou (2012)	1588	6.4/‐	United States	
Huang et al. (2010)	288		United States	Banking
Jin et al. (2017)	4968		United States	
Li et al. (2011)	832		United States	
Li et al. (2019)	802–808	8/55	United States	Hospitality
Melinsky (2013)	218	‐	United States	
Morrissey (2002)	556		United States	
Noguera (2007)	259–325	‐	United States	Real Estates
Smirnova et al. (2017)	1338	11.73/‐	Europe	
Smith and Swan (2008)	15,611		United States	
Stammerjohan (2004)	249–435	9.2/58.2	United States	
Weber (2006)	14,447		United States	

#### 5.1.3 Excluded studies

Studies were excluded for numerous reasons per our inclusion criteria. A common reason is their focus on total compensation rather than specific financial incentives (e.g., Soloski & Martin, 2019), which are our focus in this review. Studies also were excluded when they did not report overall results for financial incentives across firms but reported only results for subgroups to test moderator effects (e.g., following different forms of CEO turnover, Blackwell et al., 2007). Another reason for exclusion is the reversed direction of effects, which we found to be widespread in the CEO compensation literature (Bulmash, 2010; Frydman & Jenter, 2010). Specifically, the empirical literature gives considerable attention to the effect of past firm financial performance on current CEO financial incentives (Tosi et al., 2000) rather than our review's focus on financial incentives and their prediction of subsequent firm financial performance. Indeed given its importance, we were surprised at how few studies looked at the predictive effects of CEO incentive compensation on firm performance, despite how widely such effects are assumed (e.g., Jensen & Murphy, 1990).

### 5.2 Risk of bias in included studies

Two raters assessed study‐level risk of bias after primary studies were identified and full‐text had been extracted. We note that all primary studies reported archival data, using relatively standard metrics for firm performance. No study explicitly discussed missing data and thus we were unable to determine whether this might have been a problem. In all cases, sample representativeness (of the population) appeared sufficient given the archival sources used. Standard industry performance metrics were used and thus appear to be sufficiently reliable. Controls typically contained a core set of variables including prior year performance, firm assets or size and industry type. Some studies but not all also included CEO demographics as controls. Few studies offered justification for the controls used or described the analyses conducted to determine the appropriateness of controls.

We planned to examine whether Risk of Bias levels affected effect sizes along with potential effects attributable to time frame, firm size, country context and methodological differences (e.g., analytic method, and the number and type of covariates). However, the small number of studies for any specific incentive x performance combination precluded testing for effects of Bias or most moderator effects. However, we conducted meta‐regression to test the effects of publication status and time frame of publication (Table 5). Meta‐regression yielded a significant effect of publication status with published studies having an average population effect size of 0.041 (*k* = 38, 95% CI = 0.012, 0.070) and unpublished studies 0.010 (*k* = 11, 95% CI = −0.042, 0.061) with the effect size estimated for unpublished studies falling below the CI for published ones. Meta‐regression analysis also suggests an increase in effect sizes in studies conducted after the 2008 financial crisis compared to those conducted before. The pre‐crisis population effect size is −0.007 (*k* = 17, 95% CI = −0.052, 0.038) while the post‐crisis effect size is 0.053 (*k* = 32, 95% CI = 0.025, 0.082).

**Table 5:** Results of the meta‐regression analysis (DV = combined).

Variable	Correlations	Population effect size	95% CI	Q	*I* ^2^(%)	Tau^2^	*z*
Performance	49 (20 studies)	0.034	[0.009, 0.059]	505.10***	94.71	0.0065	2.67***
Restatement	4 (3 studies)	−0.090	[−0.363, 0.184]	67.83***	98.82	0.0760	−0.64

Subgroups	Correlations	Population effect size	95% CI	Q	*I* ^2^(%)	Tau^2^	H^2^
Lagged time = 1‐year	39 (20 studies)	0.045	[0.017, 0.073]	477.69***	95.81	0.007	23.84
Lagged time = 3‐year	6 (3 studies)	−0.015	[−0.087, 0.057]	15.45**	69.54	0.006	3.28
Lagged time = 5‐year	4 (2 studies)	−0.007	[−0.083, 0.068]	7.20	60.37	0.003	2.52
Before 2008 financial crisis	17 (7 studies)	−0.007	[−0.052, 0.038]	309.50***	95.30	0.007	21.28
After 2008 financial crisis	32 (13 studies)	0.053	[0.025, 0.082]	188.25***	92.97	0.005	14.23
Unpublished	11 (6 studies)	0.010	[−0.042, 0.061]	92.27***	91.94	.006	12.41
Published	38 (14 studies)	0.041	[0.012, 0.070]	268.18***	94.90	0.007	19.61
Bonus‐ROA	10 (7 studies)	0.041	[0.015, 0.068]	43.19***	67.77	0.001	3.10
Bonus‐Tobin's Q	3 (3 studies)	0.028	[−0.024, 0.080]	36.07***	93.09	0.002	14.47
Bonus‐Stock Return	8 (5 studies)	−0.052	[−0.122, 0.017]	33.84***	76.63	0.007	4.28
Bonus‐etc	3 (3 studies)	0.002	[−0.045, 0.052]	4.41	54.98	0.001	2.22
Stock Options‐ROA	7 (5 studies)	0.035	[0.009, 0.061]	11.33	50.08	0.001	2.00
Stock Options‐Tobin's Q	5 (5 studies)	0.097	[−0.027, 0.220]	47.06***	98.71	0.019	77.24
Stock Options‐Stock Return	8 (6 studies)	0.023	[−0.042, 0.088]	59.91***	87.80	0.007	8.20
Stock Options‐etc	2 (2 studies)	0.145	[−0.080, 0.371]	7.84**	87.24	0.023	7.84

** All of subgroups with *k* = 1 are dropped.

*** *p* < 0.001.

#### 5.2.1 Allocation (selection bias)

N/A.

#### 5.2.2 Blinding (performance bias and detection bias)

N/A All studies are based on archival records.

#### 5.2.3 Incomplete outcome data (attrition bias)

N/A All studies are based on archival records of publicly reported information.

#### 5.2.4 Selective reporting (reporting bias)

N/A.

#### 5.2.5 Other potential sources of bias

N/A.

### 5.3 Effects of interventions

Our final set of included articles contains 20 articles (a total of 49 effect sizes) pertinent to our first question, whether CEO financial incentives predict firm performance and 3 articles (a total of 4 effect sizes) pertinent to our second question, whether CEO financial incentives predict a firm's financial restatement (see Table 5).

#### 5.3.1 Question 1: CEO financial incentives and firm financial performance

A total of 20 studies (15,398 firms) are included in our analysis of CEO incentives as predictors of firm financial performance. Although we extracted effect sizes for 1 (20 studies), 2 (3 studies) and 3 (2 studies) year lags between CEO incentive granting and financial result (Tables 6, 7, 8), we interpret here *only results for 1 year lags* (Table 6, 39 effect sizes), given the limited number of studies (*k* ≤ 2) containing longer lags for any combination of financial incentive and performance outcome.

**Table 6:** Results of the meta‐analysis (performance 1‐year lagged).

Variables	Correlations	Population effect size	95% CI	Q	*I* ^2^(%)	Tau^2^	*Z*
Lagged time = 1‐year (Subgroups with k = 1 included)	39 (20 studies)	0.045	[0.017, 0.073]	477.69***	95.81	0.007	3.11**

Subgroups	Correlations	Population effect size	95% CI	Q	*I* ^2^(%)	Tau^2^	H^2^
Bonus‐ROA	7 (7 studies)	0.046	[0.014, 0.078]	40.48***	77.39	0.001	4.42
Bonus‐Tobin's Q	3 (3 studies)	0.028	[−0.024, 0.080]	36.07***	93.09	0.002	14.47
Bonus‐Stock Return	5 (5 studies)	−0.026	[−0.119, 0.067]	23.21***	83.08	0.009	5.91
Bonus‐etc	3 (3 studies)	0.003	[−0.045, 0.052]	4.41	54.98	0.001	2.22
Stock Options‐ROA	5 (5 studies)	0.027	[−0.000, 0.053]	8.14	49.50	0.000	1.98
Stock Options‐Tobin's Q	5 (5 studies)	0.097	[−0.027, 0.220]	47.06***	98.71	0.019	77.24
Stock Options‐Stock Return	6 (6 studies)	0.042	[−0.033, 0.117]	56.85**	90.11	0.007	10.11
Stock Options‐etc	2 (2 studies)	0.145	[−0.080, 0.371]	7.84**	87.24	0.023	7.84

** All of subgroups with *k* = 1 are dropped.

*** *p* < 0.001.

**Table 7:** Results of the meta‐analysis (performance 3‐year lagged).

Variables	Correlations	Population Effect Size	95% CI	Q	*I* ^2^(%)	Tau^2^	*z*
Lagged time = 3‐year	6 (3 studies)	−0.015	[−0.087, 0.057]	15.45**	69.54	0.006	−0.41

Variables	Correlations	Population Effect Size	95% CI	Q	*I* ^2^(%)	Tau^2^	H^2^
Bonus‐ROA	2 (2 studies)	0.029	[−0.034, 0.092]	0.00	0.00	0.000	1.00
Bonus‐Stock Return	2 (2 studies)	−0.137	[−0.219, −0.056]	0.26	0.02	0.000	1.00

** All of subgroups with *k* = 1 are dropped.

**Table 8:** Results of the meta‐analysis (performance 5‐year lagged).

Variables	Correlations	Population Effect Size	95% CI	Q	*I* ^2^(%)	Tau^2^	*z*
Lagged time = 5‐year	4 (2 studies)	−0.007	[−0.083, 0.068]	7.20	60.37	0.003	2.52

##### Bonuses

Bonuses were the most common financial incentive used in our included studies. Predicting 1 year‐lagged ROA, the effect size is 0.046 (*k* = 7, 95% CI = 0.014, 0.078) indicating a small positive effect. For Stock Returns, the effect size is −0.026 which falls into a CI including 0 (*k* = 5, 95% CI = −0.119, 0.067), where 0 was the modal effect size, hence no effect is evident. For Market‐To‐Book Value (Tobin's Q), the effect size is 0.028 (*k* = 3, 95% CI = −0.024, 0.080), also with a CI including 0, suggesting no effect.

##### Stock options

Stock options were the second most common financial incentive used in our included studies with a 1‐year lag. Predicting ROA, the effect size is 0.027 (*k* = 5, 0.95% CI = −0.000, 0.052). However, we note variability, with 5 studies reporting effects between 0.0 and 0.04, and an outlier in one study of 0.11. Predicting Tobin's Q, the effect size is 0.097 (*k* = 5, 95% CI = −0.027, 0.220), with two studies having an effect size of 0 and an outlier in one study of 0.38. Predicting Stock Return, the effect size is 0.042 (*k* = 6, −0.033, 0.117) with near 0 effects in four studies and outliers in opposite directions in two studies, −0.07 and 0.17. We note that Webinger (2011), not included in our meta‐analytic results due to its incomplete statistical reporting, finds a negative effect on market returns from Stock Options but a positive effect of CEO equity holdings, reinforcing results of the present meta‐analysis that Stock Options in themselves do not to predict gains in market‐related outcomes.

#### 5.3.2 Question 2: CEO financial incentives and restatement

Three studies (*n* = 2044 firms) tested the effect of CEO financial incentives on financial restatement (Tables 5 and 9). Overall the combined effect size across financial incentives is −0.09 (*k* = 3, 95% CI = −0.363, 0.184) (0.40 [*k* = 2, 95% CI = −0.019, 0.100] for 1‐year lagged studies). For the one specific financial incentive that could be tested, the overall effect size for bonuses is −0.156 (*k* = 2, 95% CI = −0.502, 0.190). Armstrong et al. (2010) using a propensity matching design to assess the effects of CEO equity holdings, including Stock Options, provide additional evidence that CEO equity holdings do not increase accounting irregularities; moreover, they report a tendency for CEO equity holdings to reduce the likelihood of misreporting and restatement. From this admittedly small number of studies, we conclude that there is no evidence that CEO financial incentives increase the likelihood of financial restatement.

**Table 9:** Results of the meta‐analysis (DV = restatement).

Variables	Correlations	Population effect size	95% CI	Q	*I* ^2^(%)	Tau^2^	*z*
Lagged time = 1‐year	3 (2 studies)	0.040	[−0.019, 0.100]	8.28*	74.78	0.002	2.96

Subgroups	Correlations	Population effect size	95% CI	Q	*I* ^2^(%)	Tau^2^	H^2^
Bonus‐Restatement	3 (2 studies)	−0.156	[−0.502, 0.190]	58.38***	98.87	0.092	88.47

* *p* < 0.05.

*** *p* < 0.001.

## 6 DISCUSSION

Our systematic review provides modest support for the use of bonuses, given their small effect size, as a CEO financial incentive promoting the accounting performance metric ROA. In contrast, stock options, the other commonly studied incentive, have no observed effect on ROA. Moreover, we find no predictive effect of either bonuses or stock options on market‐related performance metrics. At the same time, the reliance of existing studies on archival data means that empirical research provides little to no information regarding the actual terms of the CEO's incentive contract. That is, we do not know what performance metrics might have been targeted in the CEO's contract to obtain bonuses or stock options. Past researchers have noted that connections between accounting and market‐related performance metrics cannot be assumed (Gentry & Schen, 2010). Our findings suggest that additional skepticism is warranted about the broad benefit of CEO financial incentives for the firm's stakeholders as they are currently implemented.

### 6.1 Summary of main results

1. Bonuses granted to CEOs forecast next year's ROA, but not next year's Stock Returns or Market‐to‐Book Value (Tobin's Q).
2. Stock options granted to CEOs are not predictive of next year's firm performance, neither next year's ROA nor its market‐related metrics Stock Returns and Market‐to‐Book Value.
3. Neither CEO bonuses nor stock options are related to subsequent financial restatement.

### 6.2 Overall completeness and applicability of evidence

This systematic review comprehensively searched both published and unpublished data bases, providing a thorough review of existing research over the past 40+ years. Evidence is limited to a few financial accounting measures and market‐based metrics as reflected in existing primary studies.

### 6.3 Quality of the evidence

As noted, despite the public debate regarding CEO compensation, it is noteworthy that the amount and quality of evidence is limited with respect to the predictive power or effects of incentive‐based compensation for CEOs, that is, 20 studies in 40+ years. Our review indeed finds a variety of studies examining reverse causation, past firm performance and current CEO compensation including incentives (Bulmash, 2010) where results are interpreted to infer that incentives improve future firm performance. Our findings beg the question whether potential impact on firm outcomes may be a socially acceptable rationale for CEO compensation arrangements rather than an evidence‐based practice.

Although we were not able to test contextual moderators of financial incentive effects, we note that Li and Yu (2011) find evidence that size moderates the effect of financial incentives. Specifically they find in a study of American non‐financial firms from 1993 to 2005 that small firms show a positive relationship between CEO stock‐based compensation and market returns while large firms show a negative relationship. They used a panel threshold model to test effects across various firm‐size conditions, concluding that the principle‐agent problem of incentivizing CEOs to act in shareholder interests is solved for small firms via stock‐based pay but undermined for larger firms. However, replication of such an effect is necessary as is research on the mechanisms underlying CEO behavior in response to various incentive schemes in large and small firms.

In appraising the trustworthiness of the evidence identified in the empirical literature, we conclude that publication bias favoring significant effects and the absence of information in reports on missing data and alternative analyses create moderate risk of bias in the studies we reviewed. Moreover, the possibility of non‐linear and moderating effects of CEO financial incentives in the prediction of firm performance should be considered in future reviews as the body of research literature grows.

### 6.4 Potential biases in the review process

A few studies more nuanced in their analyses than our review of direct CEO incentive‐firm financial performance effects may provide useful insight. Wang et al. (2012) used a neural network model and compared firms with best and worst performance, concluding that incentives plans are accurately associated with firm performance more than 70% of the time for best and worst firms. Chan et al. (2014) used a quantile regression analysis of CEO incentive pay in American commercial banks, reporting non‐linear relationships. They report that CEO incentive compensation improves the performance of high‐performing (top quantile) banks, which they refer to as a “carrot” approach, although it does not for banks in lower‐quintiles. They further advocate that at lower levels of performance a firm's performance may be improved by a “stick” approach where outside directors apply monitoring. Since both studies used empirical methods to identify effects without formally testing hypotheses, we hesitate to rely on their findings without replication. We also note that moderator effects may exist regarding the CEO incentive compensation—firm performance connection, as in the case of the Li and Yu (2011) investigation of firm size.

### 6.5 Agreements and disagreements with other studies or reviews

No other systematic reviews exist on either question.

## 7 AUTHORS' CONCLUSIONS

### 7.1 Implications for practice

We find that CEO financial incentives vary in whether they predict future firm performance. Bonuses have a small predictive effect on next year's ROA, while stock options do not. In addition, neither incentive predicts next year's market‐valuation of the firm (Tobin's Q) or stock returns. Despite the widespread emphasis on CEO financial incentives as a driver of firm performance (Jensen & Murphy, 1990), our findings suggest it may be problematic to justify present CEO compensation arrangements based on anticipated market results. Several factors are likely to influence our findings.

Actual CEO influence over market results or the lack of it (Bertrand & Mullainathan, 2001) is one factor, where prevailing market forces can affect firm performance to such an extent that CEOs may exercise relatively little direct control over a firm's financial outcomes. Such might be the case in market downturns or otherwise unfavorable market conditions. Conditions under which CEO exercise influence over their firm's financial outcomes may not as yet be well understood and warrant both empirical research and a future review of pertinent studies.

CEO power in shaping the incentive contract (e.g., Amzaleg et al., 2014) is a second factor, where powerful CEOs have been shown to negotiate contracts favorable to their financial gain based on anticipated (high/low) firm performance levels. CEO power can thus potentialy obscure or undermine any real effect of financial incentives on firm performance.

CEO compensation on its own may be a poor indicator of the efficiency of incentives since it is not merely the CEO who contributes to firm performance. Directing efforts more effectively toward increasing firm performance can involve appropriate compensation to the top management team (TMT) as a whole (including the CEO) and more broadly to organization members including rank and file employees. Research on the portion of total TMT compensation that is granted to the CEO (referred to as “pay slice”) finds that pay slice correlates negatively with firm financial performance while more comparability in TMT pay relates to better outcomes (Bebchuk et al., 2011). A further consideration in compensation within a firm is the ratio of CEO to employee compensation (Bao et al., 2020) and the extent to which employees broadly participate in equity ownership in the firm (Blasi & Kruse, 2000). The latter research on equity ownership by employees suggests that any equity ownership by employees provides benefits, an effect known as a “floor” effect.

The possibility of a floor effect in the context of CEOs has been examined with research findings suggesting that requiring a minimum of stock ownership may be a more effective CEO incentive than increasing the actual number of CEO‐held stock shares. Core and Larker (2002) find that a minimum of CEO stock ownership is positively related to firm financial performance, in line with findings from research on employee ownership (Blasi & Kruse, 2000; Freeman et al., 2010) where it is the condition of a minimum level of employee ownership participation rather than its extent that predicts positive firm financial performance. Applying such a condition of ownership participation to CEOs might achieve the effect sought from CEO financial incentives without producing the wealth transfers associated with greater stock participation for CEOs absent concomitant performance gains (Sanders, 2001).

Although incentivizing firm performance is the frequent rationale for increases in CEO financial incentives (Jensen & Murphy, 1990), a less widely discussed motive is CEO retention (Fulmer, 2009). This alternative motivation for CEO pay increases can undermine any potential effects on future performance if the amount and kinds of CEO pay are largely based on comparisons with how other firms pay their CEOs. If market rates set the standard for CEO compensation, connections between CEO incentive compensation and firm performance may be weakened. No study we reviewed included information about the targets specified with regard to incentive compensation. Targets set low enough to be readily met may do little to improve firm outcomes.

A final consideration we raise is whether incentivizing market results in themselves is sufficient to motivate CEO effectiveness. CEO leadership style, as opposed to compensation per se, has been shown to affect firm performance with its effects partially mediated by employee satisfaction (e.g., Wang et al., 2011; Xi et al., 2017). To the extent that employee satisfaction and other indicators of the quality of employment relationships affect firm financial outcomes, employee satisfaction may be an appropriate metric to include in evaluating CEO effectiveness. Along these lines, Edmans (2011) finds that in a study of “100 Best Companies to Work for in America” that while employee satisfaction is positively related to shareholder returns, the stock market does not fully value such intangibles, and recommends screening on socially responsible investing to improve investment returns. One factor in employee satisfaction is the ratio of CEO to employee compensation (Bao et al., 2020; Rouen, 2020) as addressed above. Research findings show that a higher CEO/employee pay ratio based on economic factors such as firm size yields better firm outcomes than does a higher ratio based on non‐economic factors like CEO power (Bao et al., 2020). Such findings suggest that comparative pay studies with comparable firms can provide insight regarding appropriate CEO compensation that in turn better motivates member contributions to improve firm financial results. Such a comparative factor may be firm size, which effects both controllability of outcomes by CEO as well as the processes whereby CEO influence might occur (cf. Li & Yu, 2011).

In sum, review findings modestly support CEO compensation contracts that use bonuses to target increased ROA. Findings do not support reliance on CEO financial incentives to improve future firm market‐related performance. For compensation committees and other firm stakeholders seeking to develop effective CEO compensation contracts, we call attention to the floor effect of equity, such that CEOs (Core & Larker, 2002) and employees (Blasi & Kruse, 2000) with at least some equity stake are likely to be motivated to direct efforts toward the organization's success, an effect that need not increase as equity increases.

### 7.2 Implications for research

Having searched for primary studies examining CEO financial incentives and firm performance, we note that several studies identified in our initial screening focused not on the predictive effect of CEO incentives on firm outcomes but the reverse. Several of these studies made claims about the impact of CEO incentives on firm performance while actually testing for the opposite, that is, the effect of firm performance on CEO financial incentives (e.g., Bulmash, 2010). The focus on this reverse effect, couched in the language of CEO influence over firm performance, is often difficult to discern without close reading of a primary study's method section. Other studies test the relationship in both directions but pay less attention to controlling for past performance to test predictive effects (e.g., Zandi et al., 2019).

We note limited attention in the primary studies we identified in this review to shareholder return, as indicated by stock returns (i.e., change in share price over the year plus dividends divided by beginning of year price). Only 5 studies identified for inclusion considered this outcome. This limited attention to shareholder returns is noteworthy because of the importance assigned to shareholder interests in the economics literature (e.g., Jensen & Murphy, 1990). We note that Haynes et al. (2017) report a negative relationship between shareholder returns and CEO greed, where greed is operationalized using three proxy indicators: (1) Market reaction to the form of CEO compensation, (2) comparison to other top executives in the firm (i.e., pay slice), and (3) comparison of total CEO pay to known predictors in extant research such as the economic factors mentioned above, an indicator of overpayment. Greed aligns with CEO influence over their pay levels, and is negatively related to shareholder return. Effects of CEO greed on shareholder returns are mitigated by a powerful independent board (which reduces the effect of greed as board independence increases), managerial discretion (which increases the effect of greed as it increases) and CEO tenure (which reduces the effect of greed as tenure increases).

Previous research by Gentry and Shen (2010) found that accounting and market measures of firm financial performance are largely independent. Our findings of different effects for ROA and market‐related measures is in line with their findings. How these two kinds of metrics are connected has been examined in an investigation of how CEO option wealth influences the efforts made regarding firm productivity and subsequent market value (Zolotoy et al., 2022). That study offers empirical evidence of how options might affect CEO behavior and resulting firm outcomes by considering the CEO wealth at stake in the firm's performance. This more nuanced specification of how options affect CEO effort and attention warrants further research.

With respect to the relationship of CEO incentives to financial restatement, we found only three studies focused on financial restatement as an outcome and CEO incentives as a predictor with varying effect sizes and a CI containing 0. This finding has a variety of implications. First, future research should consider whether it is CEOs themselves that are the likely perpetrators of fraud or a broader array of executives, including Chief Financial Officers. In this regard Lux et al. (2023) investigated the compensation structures of CEOs and CFOs (unfortunately for our purposes conflating the two), finding differences between fraud and non‐fraud firms in executive stock and option awards (though not bonuses). They also find that delayed compensation (including stock and option awards) is greater in the non‐fraud group. They also noted that executives in the non‐fraud group tended to be paid more on average. Second, we suggest that a simple model of CEO incentives ‐‐> fraud including financial restatement may be inadequate to account for any link between CEO compensation and financial misreporting. The level and composition of compensation packages may also be a factor, as these contribute to the renumeration goals CEOs (and other executives) may seek to attain.

Last, from a research perspective, we suggest that future research investigate the black box of CEO financial incentive‐to‐firm performance connections. By this we mean how actual executives “think” about the incentives applied to them and whether there is indeed a set of consistent effects for particular types of incentive arrangements on CEO beliefs and behavior. Many assumptions have been made regarding how executives should think and act under various incentive conditions without actual empirical investigation of the judgments and actions they indeed display. Research that investigates the judgments managers make in determining how to act can use the methodology of policy capturing (Karrin & Barringer, 2002; Tomprou et al., 2023; Wang et al., 2015), where the factors influencing an individual's judgments (referred to as “policies”) are empirically tested. Policy capturing research is recommended to investigate the kinds of priorities and considerations that might be generated by different executive incentive arrangements.

## CONTRIBUTIONS OF AUTHORS

Denise Rousseau, an organizational psychologist, was primarily responsible for writing the protocol and final report. Responsibilities included formulating the overview of the project, interpreting relevant literature in problem framing, and assisting in identifying relevant concepts to construct our PICO. Rousseau also screened studies, and participated in the extraction of and interpretation of results. Our team librarians, Donna Beck, Ryan Splenda and Sarah Young developed the search strategy and identified appropriate search terms. Ryan Splenda, a business librarian, contributed to the formulation of the outcome measures appropriate for addressing our question. Sarah Young, developed our extraction documentation. Librarians Splenda and Young facilitated the search and coding process by inputing primary studies into Covidence for screening and extraction. ByeongJo Kim, a management scholar, assisted in literature reviews as we prepared our search strategy. Rousseau and Kim coded the studies and resolved conflicting codes. Dr. Kim and Mr. Lee, a doctoral student, performed the statistical analyses summarized in Findings and constructed results tables.

## DECLARATIONS OF INTEREST

No team member has a financial interest related to the topic of CEO incentive pay. This review was not supported by a funding agency or other entity outside our universities.

## DIFFERENCES BETWEEN PROTOCOL AND REVIEW

Given the small number of studies identified for each incentive X performance combination, we are unable to assess the effects of different sets of controls or the moderating effects of CEO tenure or age. Similarly, with regard to assessments of bias, we observe that the primary studies investigating effects on firm performance virtually never reported on missing data, judgments made regarding data inclusion or use of controls, and analyses conducted not included in the final report. These omissions exacerbate risk of bias. As such we estimate the Risk of Bias as moderate across all studies but cannot test for its effects on results.

## PUBLISHED NOTES

## CHARACTERISTICS OF STUDIES

**Characteristics of included studies**

*Footnotes*

**Characteristics of excluded studies**

*Footnotes*

**Characteristics of studies awaiting classification**

*Footnotes*

**Characteristics of ongoing studies**

*Footnotes*

**Summary of findings tables**

Additional tables

## DATA AND ANALYSES

## SOURCES OF SUPPORT

**Internal sources**

- Heinz II Chair, USA.

Funding for first author.

**External sources**

- No sources of support provided.

Feedback

## Supporting information

Supporting information.
