# Supplementary material for: Does chief executive compensation predict financial performance or inaccurate financial reporting in listed companies: A systematic review
Source: Campbell Syst Rev. 2023 Dec 11;19(4):e1370. doi: 10.1002/cl2.1370 (PMC10712439; doi:10.1002/cl2.1370)
Supplement: Supplementary file 1 — Supporting information. [file CL2-19-e1370-s001.docx]

Appendices

## 1 ABI/INFORM search strategy

| 1 | MAINSUBJECT(“Chief executive officer*“) |
| --- | --- |
| 2 | AB,TI(ceo OR ceos OR “chief executive officer*“ OR “senior leader*“ OR “corporate director*” OR executive*) |
| 3 | 1 OR 2 |
| 4 | MAINSUBJECT(Bonus* OR “monetary incentive*” OR “productivity incentive*” OR compensat* OR “compensat* plans” OR “incentive plan*” OR “pay for performance” OR “unreasonable compensation” OR “stock option*” OR “executive compensation”) |
| 5 | AB,TI(bonus* OR incentiv* OR “pay for performance” OR compensation OR salary OR salaries OR “stock options” OR “performance award*” OR “performance based pay” OR “performance pay” OR “performance-related pay” OR “executive pay” OR “executive profit sharing”) |
| 6 | 4 OR 5 |
| 7 | MAINSUBJECT.EXACT(“financial performance” OR “corporate profits” OR earnings OR “retained earnings” OR “undistributed profits” OR “earnings per share” OR “return on investment” OR revenue OR “financial ratios” OR “return on assets” OR “return on equity” OR “corporate mergers” OR “market value”) |
| 8 | AB,TI(firm* OR corporate OR corporation* OR company OR companies OR organizational OR financial OR business) NEAR/5 AB,TI(performance OR profit* OR “stock price” OR value OR sales OR revenue OR “market share” OR innovation OR growth OR “financial health” OR liquidity) |
| 9 | AB,TI(“return on assets” OR ROA OR “return on income” OR ROI OR ROIC OR “return on invested capital” OR “return on capital” OR ROC OR “return on equity” OR ROE OR EBITDA OR “earnings before interest tax depreciation and amortization” OR “stock market returns” OR “equity pricing” OR “profit margin” OR “financial ratio” OR “merger and acquisition” OR “mergers and acquisitions” OR “market value” OR “market return*“ OR “market to book” OR “price to book” OR “total shareholder return”) |
| 10 | 7 OR 8 OR 9 |
| 11 | MAINSUBJECT.EXACT(“financial restatements” OR “accounting irregularities”) |
| 12 | AB,TI(financ* OR account* OR corporate) NEAR/5 AB,TI(misrepresent* OR restatement* OR mislead* OR negligen* OR irregular* OR inaccura* OR inconsisten* OR manipulate* OR decept* OR dishonest OR deceive* OR misreport* OR fraud* OR lying OR false) |
| 13 | 11 OR 12 |
| 14 | 10 OR 13 |
| 15 | 3 AND 6 AND 14 |
| 16 | Limits: Source Type—Scholarly Journals, Dissertations & Theses, Working Papers, Conference Papers & Proceedings |
| 17 | Limit: 1980-01-01 to present |

## 2 Data Extraction Form

The following fields will be used to code and extract data from each article. All data will be initially coded in Excel for tracking purposes and warehousing information.

**I. Relevance Screening**

a.Document Full Citation (APA style)

b. Is the document about a longitudinal study?

⎕ Yes

⎕ Unclear

⎕ No (explain) – STOP REVIEW

c. Timing: Was the study reported post January 1980?

⎕ Yes (initial year of data collection)?

⎕ No (explain) – STOP REVIEW

d. Does the study examine CEO incentive contracts?

⎕ Yes

⎕ No – STOP REVIEW

e. Does the study include a metric of firm performance or financial restatements measured after CEO incentive contracts?

⎕ Yes

⎕ No – STOP REVIEW

f. Does the study include publically traded corporations?

⎕ Yes

⎕ No – STOP REVIEW

g. Does the study include controls for a) pre-incentive firm performance and/or b) market conditions prevailing at the time?

⎕ Yes

⎕ No – STOP REVIEW

II. **Data Extraction Worksheet**

**Study ID:**

**Coder Name:**

**Study description**

a. How many documents are associated with this study?

b. Type of publication [for each study document]

⎕ Book

⎕ Peer-reviewed journal article

⎕ Book chapter (in an edited book)

⎕ Thesis or dissertation

⎕ Technical report

⎕ Conference paper

⎕ Other:___________________

⎕ Unreported/cannot tell

c. Countries in which study was conducted [check all that apply]

⎕ USA

⎕ Canada

⎕ Great Britain

⎕ Other English speaking

⎕ Other ____________________

⎕ Unreported/cannot tell

d. Industries in which study was conducted

⎕ Diverse industries of publicly traded firms

⎕ Industry subset of publicly traded firms? Which?____________

e. Sample size - number of firms included in the study

f. Funding sources, if any (for the study)

**Study methods**

a. Were comparison groups included?

⎕ Yes [explain nature of comparisons, e.g., CEO incentive versus no incentive, or comparison of different types of incentives]

⎕ No

**Firm characteristics**

a. number of employees (minimum, maximum, mean, standard deviation)

b. number of sites/locations per firm (min, max, mean, sd)

c. annual revenue (min, max, mean, sd)

**CEO characteristics**

a, age (min, max, mean, sd)

b. tenure [years in position] (min, max, mean, sd)

c, gender [% in subgroups]

d. race/ethcnicity [% in subgroups]

e. base salary (min, max, mean, sd)

**CEO incentive schemes**

a. What types of incentives were studied [check all that apply]

⎕ Cash bonus

⎕ Stock options

⎕ Salary increase

⎕ Other [explain]

b. What targets were tied to incentives [check all that apply]

⎕ Stock price

⎕ Revenue growth

⎕ Other [explain]

**Firm performance measures**

a. What performance measures were included? [check all that apply; for each, explain how the construct was operationalized and how [and by whom] measures were created/obtained}

⎕ Profitability: Return on investment (ROI)

⎕ Profitability: Return on Assets (ROA)

⎕ Profitability: Return on Assets (ROIC)

⎕ Profitability: Return on Assets (ROC)

⎕ Profitability: Return on Assets (ROE)

⎕ Profitability: Return on Assets (EBITDA)

⎕ Market returns: Market-to-book value

⎕ Other indicators of increased shareholder returns

⎕ Other [explain]

⎕ None

b. Timing of performance measures available [check all that apply]

⎕ prior to incentive contract

⎕ at the time of the incentive contract

⎕ within one year after the contract

⎕ >1-2 years after

⎕ >2-3 years

⎕ >4-5 years

⎕ more than 5 years

⎕ Not applicable

**Financial restatement measures**

a. Are financial restatement indicators available?

⎕ Yes - explain how [and by whom] restatement indicators were created/obtained

⎕ No

**Analytic methods**

a. Statistical methods used [check all that apply]

⎕ bivariate associations

⎕ multivariate models - identify types of models used

⎕ other [explain]

b. Statistics reported [check all that apply]

⎕ correlation coefficients (unadjusted)

⎕ partial correlations

⎕ regression coefficients

⎕ other [explain]

c. Covariates used in regression (or other multivariate) models [number and type of covariates]

d. Was data imputation used to fill in missing data?

⎕ Yes: Describe imputation methods used _____________

⎕ No

⎕ Unclear

**Results**

a. For each relevant analysis in the study, we will extract data on

⎕ type of CEO incentive and target

⎕ nature and timing of outcome measures (performance indicators or financial restatement)

⎕ valid n

⎕ type of effect size [r, partial r, b or other]

⎕ values of effect sizes

⎕ values of SE or other measures of variability [e.g., confidence interval]

⎕ number and types of covariates in the model, if any

⎕ p-values

⎕ method of imputation, if any

**III. Risk of Bias Assessment**

a. Selection bias: Nonequivalent comparison groups

⎕ Low risk [comparison groups were equivalent at baseline, i.e., no initial differences between CEOs with and without incentive contracts]

⎕ High risk [comparison groups exhibited differences at baseline]

⎕ Unclear

⎕ Not applicable [comparison groups were not used]

b. Selection bias: Representativeness

⎕ Low risk [representative sample of larger population of firms]

⎕ High risk [convenience sample of firms]

⎕ Unclear

c. Missing data: Attrition bias

⎕ Low risk [results for all firms are fully reported]

⎕ High risk [incomplete outcome data for some or all firms, e.g., missing data on financial restatements]

⎕ Unclear

d.Missing data: Outcome reporting bias

⎕ Low risk [outcomes are fully reported regardless of direction and significance of results]

⎕ High risk [statsitically non-significant results are not fully reported, e.g., missing r or SE or valid n]

⎕ Unclear

e. Detection bias: Unreliability of measures

⎕ Low risk [all measures are judged to be reliable and valid]

⎕ High risk [some or all outcome measures appear to be unreliable or invalid]

⎕ Unclear

f. Other sources of bias: Lack of adequate controls for relevant confounders

⎕ Low risk [relevant confounders are included as controls in analysis]

⎕ High risk

⎕ Unclear

## 3 Supplementary File 1

## Supplementary File 1. Search Strategy

**Methodology**

The search syntax was created and adapted for each source listed. We primarily focused and executed our searches using the title, abstract, and subject indexing fields (when appropriate and available). The full search strategies and results for each resource is provided below. We used available filters to remove news articles, trade journals, industry reports, and magazine articles that are not relevant to our study. These searches capture research that was published until December of 2019. Our team’s information specialists (RS, SY) ran update searches within these resources in July of 2021 to capture any new studies that were published since December of 2019. The same search strategies and their adaptations were used in all of the resources when the update was performed.

### Database searches

**Database:** ABI/INFORM

**Platform:** ProQuest

**Date of Search:** December 13, 2019 (updated July 23, 2021)

| 1 | MAINSUBJECT(“Chief executive officer*“) | 1,276,816 |
| --- | --- | --- |
| 2 | AB,TI(ceo OR ceos OR “chief executive officer*“ OR “senior leader*“ OR “corporate director*“ OR executive*) | 3,732,040 |
| 3 | 1 OR 2 | 4,600,809 |
| 4 | MAINSUBJECT(Bonus* OR “monetary incentive*“ OR “productivity incentive*“ OR compensat* OR “compensat* plans” OR “incentive plan*“ OR “pay for performance” OR “unreasonable compensation” OR “stock option*“ OR “executive compensation”) | 576,330 |
| 5 | AB,TI(bonus* OR incentiv* OR “pay for performance” OR compensation OR salary OR salaries OR “stock options” OR “performance award*“ OR “performance based pay” OR “performance pay” OR “performance-related pay” OR “executive pay” OR “executive profit sharing”) | 957,848 |
| 6 | 4 OR 5 | 1,385,032 |
| 7 | MAINSUBJECT.EXACT(“financial performance” OR “corporate profits” OR earnings OR “retained earnings” OR “undistributed profits” OR “earnings per share” OR “return on investment” OR revenue OR “financial ratios” OR “return on assets” OR “return on equity” OR “corporate mergers”) | 8,322,243 |
| 8 | AB,TI(firm* OR corporate OR corporation* OR company OR companies OR organizational OR financial OR business) NEAR/5 AB,TI(performance OR profit* OR “stock price” OR value OR sales OR revenue OR “market share” OR innovation OR growth OR “financial health” OR liquidity) | 1,946,432 |
| 9 | AB,TI(“return on assets” OR ROA OR “return on income” OR ROI OR ROIC OR “return on invested capital” OR “return on capital” OR ROC OR “return on equity” OR ROE OR EBITDA OR “earnings before interest tax depreciation and amortization” OR “stock market returns” OR “equity pricing” OR “profit margin” OR “financial ratio” OR “merger and acquisition” OR “mergers and acquisitions” OR “earnings per share”) | 1,053,239 |
| 10 | 7 OR 8 OR 9 | 10,399,702 |
| 11 | MAINSUBJECT.EXACT(“financial restatements” OR “accounting irregularities”) | 2,438 |
| 12 | AB,TI(financ* OR account* OR corporate) NEAR/5 AB,TI(misrepresent* OR restatement* OR mislead* OR negligen* OR irregular* OR inaccura* OR inconsisten* OR manipulate* OR decept* OR dishonest OR deceive* OR misreport* OR fraud* OR lying OR false) | 41,793 |
| 13 | 11 OR 12 | 43,626 |
| 14 | 10 OR 13 | 10,437,186 |
| 15 | 3 AND 6 AND 14 | 28,707 |
| 16 | Limits: Source Type—Scholarly Journals, Dissertations & Theses, Working Papers, Conference Papers & Proceedings | 2,634 |
| 17 | Limit: 1980-01-01 to 2020 | 2,609 |

**Database:** Web of Science Core Collection (Social Sciences Citation Index (SSCI), Science Citation Index Expanded (SCI-EXPANDED), Conference Proceedings Citation Index – Science (CPCI-S), Conference Proceedings Citation Index – Social Science & Humanities (CPCI-SSH), Emerging Sources Citation Index (ESCI)

**Platform:** Clarivate Analytics

**Date of Search:** December 13, 2019 (updated July 23, 2021)

| 1 | TS=(ceo OR ceos OR “chief executive officer*“ OR “senior leader*“ OR “corporate director” OR “corporate directors” OR “corporate directorships” OR executive*) | 107,557 |
| --- | --- | --- |
| 2 | TS=(bonus* OR incentiv* OR “pay for performance” OR compensation OR salary OR salaries OR “stock options” OR “performance award” OR “performance awards” OR “performance based pay” OR “performance pay” OR “performance-related pay” OR “executive pay” OR “executive profit sharing”) | 316,007 |
| 3 | TS=((firm* OR corporate OR corporation* OR company OR companies OR organizational OR financial OR business) NEAR/5 (performance OR profit* OR “stock price” OR value OR sales OR revenue OR “market share” OR innovation OR growth OR “financial health” OR liquidity)) | 151,215 |
| 4 | TS=(“return on assets” OR ROA OR “return on income” OR ROI OR ROIC OR “return on invested capital” OR “return on capital” OR ROC OR “return on equity” OR ROE OR EBITDA OR “earnings before interest tax depreciation and amortization” OR “stock market returns” OR “equity pricing” OR “profit margin” OR “financial ratio” OR “merger and acquisition” OR “mergers and acquisitions”) | 92,409 |
| 5 | TS=((financ* OR account* OR corporate) NEAR/5 (misrepresent* OR restatement* OR mislead* OR negligen* OR irregular* OR inaccura* OR inconsisten* OR manipulate* OR decept* OR dishonest OR deceive* OR misreport* OR fraud* OR lying OR false)) | 7,888 |
| 6 | #3 OR #4 OR #5 | 247,197 |
| 7 | #1 AND #2 AND #6 | 2,956 |
| 8 | Limit: 1980-01-01 to present | 2,950 |

**Database:** EconLit

**Platform:** EBSCO

**Date of Search:** December 13, 2019 (updated July 23, 2021)

**Limit:** 1980-01-01 to present

| 1 | TX(ceo OR ceos OR “chief executive officer*“ OR “senior leader*“ OR “corporate director*“ OR executive*) | 19610 |
| --- | --- | --- |
| 2 | TX(bonus* OR incentiv* OR “pay for performance” OR compensation OR salary OR salaries OR “stock options” OR “performance award*“ OR “performance based pay” OR “performance pay” OR “performance-related pay” OR “executive pay” OR “executive profit sharing”) | 91883 |
| 3 | TX((firm* OR corporate OR corporation* OR company OR companies OR organizational OR financial OR business) N5 (performance OR profit* OR “stock price” OR value OR sales OR revenue OR “market share” OR innovation OR growth OR “financial health” OR liquidity)) | 127657 |
| 4 | TX(“return on assets” OR ROA OR “return on income” OR ROI OR ROIC OR “return on invested capital” OR “return on capital” OR ROC OR “return on equity” OR ROE OR EBITDA OR “earnings before interest tax depreciation and amortization” OR “stock market returns” OR “equity pricing” OR “profit margin” OR “financial ratio” OR “merger and acquisition” OR “mergers and acquisitions” OR “earnings per share”) | 7772 |
| 5 | 3 OR 4 | 132191 |
| 6 | TX((financ* OR account* OR corporate) N5 (misrepresent* OR restatement* OR mislead* OR negligen* OR irregular* OR inaccura* OR inconsisten* OR manipulate* OR decept* OR dishonest OR deceive* OR misreport* OR fraud* OR lying OR false)) | 1296 |
| 7 | 5 OR 6 | 132949 |
| 8 | 1 AND 2 AND 7 | 4011 |

**Database:** Business Source Ultimate

**Platform:** EBSCO

**Date of Search:** December 13, 2019 (updated in Business Source Premier July 23, 2021)

| 1 | DE(“chief executive officers”) | 122,803 |
| --- | --- | --- |
| 2 | AB(ceo OR ceos OR “chief executive officer*“ OR “senior leader*“ OR executive* OR “corporate director*“) | 866,349 |
| 3 | TI(ceo OR ceos OR “chief executive officer*“ OR “senior leader*“ OR executive* OR “corporate director*“) | 112,847 |
| 4 | 1 OR 2 OR 3 | 910,099 |
| 5 | DE(“monetary incentives” OR “productivity incentives” OR “stock options” OR “executive compensation” OR “pay for performance” OR “unreasonable compensation”) | 26,877 |
| 6 | AB(bonus* OR incentive* OR “pay for performance” OR compensation OR salary OR salaries OR “stock options” OR “performance award” OR “performance based pay” OR “performance pay” OR “performance-related pay” OR “executive pay” OR “executive profit sharing”) | 249,408 |
| 7 | TI(bonus* OR incentive* OR “pay for performance” OR compensation OR salary OR salaries OR “stock options” OR “performance award” OR “performance based pay” OR “performance pay” OR “performance-related pay” OR “executive pay” OR “executive profit sharing”) | 56,553 |
| 8 | 5 OR 6 OR 7 | 267,304 |
| 9 | DE(“financial performance” OR “corporate profits” OR “earnings per share” OR “rate of return” OR “financial ratios” OR “return on assets” OR “consolidation & merger of corporations” OR “retained earnings”) | 579,772 |
| 10 | AB((firm* OR corporate OR corporation* OR company OR companies OR organizational OR financial OR business) N5 (performance OR profit* OR “stock price” OR value OR sales OR revenue OR “market share” OR innovation OR growth OR “financial health” OR liquidity)) | 704,085 |
| 11 | TI ((firm* OR corporate OR corporation* OR company OR companies OR organizational OR financial OR business) AND (performance OR profit* OR “stock price” OR value OR sales OR revenue OR “market share” OR innovation OR growth OR “financial health” OR liquidity)) | 57,475 |
| 12 | AB(“return on assets” OR ROA OR “return on income” OR ROI OR ROIC OR “return on invested capital” OR “return on capital” OR ROC OR “return on equity” OR ROE OR EBITDA OR “earnings before interest tax depreciation and amortization” OR “stock market returns” OR “equity pricing” OR “profit margin” OR “financial ratio” OR “merger and acquisition” OR “mergers and acquisitions” OR “earning per share”) | 60,164 |
| 13 | TI(“return on assets” OR ROA OR “return on income” OR ROI OR ROIC OR “return on invested capital” OR “return on capital” OR ROC OR “return on equity” OR ROE OR EBITDA OR “earnings before interest tax depreciation and amortization” OR “stock market returns” OR “equity pricing” OR “profit margin” OR “financial ratio” OR “merger and acquisition” OR “mergers and acquisitions”) | 8,595 |
| 14 | 9 OR 10 OR 11 OR 12 OR 13 | 1,182,535 |
| 15 | DE(“restatement of corporate earnings” OR “misleading financial statements” OR “accounting fraud”) | 3,134 |
| 16 | AB((finance* OR account* OR corporate) N5 (misrepresent* OR restatement* OR mislead* OR negligen* OR irregular* OR inaccura* OR inconsisten* OR manipulate* OR decept* OR dishonest OR deceive* OR misreport* OR fraud* OR lying OR false)) | 8,671 |
| 17 | TI((finance* OR account* OR corporate) AND (misrepresent* OR restatement* OR mislead* OR negligen* OR irregular* OR inaccura* OR inconsisten* OR manipulate* OR decept* OR dishonest OR deceive* OR misreport* OR fraud* OR lying OR false)) | 1,157 |
| 18 | 15 OR 16 OR 17 | 11,266 |
| 19 | 14 OR 18 | 1,192,553 |
| 20 | 4 AND 8 AND 19 | 1,194,028 |
| 21 | Limits: Source Type – Books, Academic Journals | 2,505 |
| 22 | Limit: 1-1-1980 to present | 2,444 |
| 23 | Limit: Book, Academic Journals | 1,863 |

**Database:** Dissertations & Theses Global

**Platform:** ProQuest

**Date of Search:** December 13, 2019 (updated July 23, 2021)

| 1 | noft(ceo OR ceos OR “chief executive officer*“ OR “senior leader*“ OR “corporate director” OR “corporate directors” OR “corporate directorships” OR executive*) | 29,282 |
| --- | --- | --- |
| 2 | noft(bonus* OR incentiv* OR “pay for performance” OR compensation OR salary OR salaries OR “stock options” OR “performance award*“ OR “performance based pay” OR “performance pay” OR “performance-related pay” OR “executive pay” OR “executive profit sharing”) | 62,667 |
| 3 | noft((firm* OR corporate OR corporation* OR company OR companies OR organizational OR financial OR business) NEAR/5 (performance OR profit* OR “stock price” OR value OR sales OR revenue OR “market share” OR innovation OR growth OR “financial health” OR liquidity)) | 54,441 |
| 4 | noft(“return on assets” OR ROA OR “return on income” OR ROI OR ROIC OR “return on invested capital” OR “return on capital” OR ROC OR “return on equity” OR ROE OR EBITDA OR “earnings before interest tax depreciation and amortization” OR “stock market returns” OR “equity pricing” OR “profit margin” OR “financial ratio*“ OR “mergers and acquisition*“ OR “earnings per share”) | 11,155 |
| 5 | noft((financ* OR account* OR corporate) NEAR/5 (misrepresent* OR restatement* OR mislead* OR negligen* OR irregular* OR inaccura* OR inconsisten* OR manipulate* OR decept* OR dishonest OR deceive* OR misreport* OR fraud* OR lying OR false)) | 2,176 |
| 6 | 3 OR 4 OR 5 | 65,220 |
| 7 | 1 AND 2 AND 6 | 1,267 |
| 8 | Limit: 1980-01-01 to 2020 | 1,261 |

**Database:** Scopus

**Platform:** Elsevier

| 1 | TITLE-ABS-KEY(ceo OR ceos OR “chief executive officer*“ OR “senior leader*“ OR “corporate director*“ OR executive*) | 172,623 |
| --- | --- | --- |
| 2 | TITLE-ABS-KEY (bonus* OR incentiv* OR “pay for performance” OR compensation OR salary OR salaries OR “stock options” OR “performance award*“ OR “performance based pay” OR “performance pay” OR “performance-related pay” OR “executive pay” OR “executive profit sharing”) | 454,274 |
| 3 | TITLE-ABS-KEY ((firm* OR corporate OR corporation* OR company OR companies OR organizational OR financial OR business) W/5 (performance OR profit* OR “stock price” OR value OR sales OR revenue OR “market share” OR innovation OR growth OR “financial health” OR liquidity)) | 256,538 |
| 4 | TITLE-ABS-KEY (“return on assets” OR ROA OR “return on income” OR ROI OR ROIC OR “return on invested capital” OR “return on capital” OR ROC OR “return on equity” OR ROE OR EBITDA OR “earnings before interest tax depreciation and amortization” OR “stock market returns” OR “equity pricing” OR “profit margin” OR “financial ratio” OR “merger and acquisition” OR “mergers and acquisitions” OR “earnings per share”) | 176,567 |
| 5 | TITLE-ABS-KEY ((financ* OR account* OR corporate) W/5 (misrepresent* OR restatement* OR mislead* OR negligen* OR irregular* OR inaccura* OR inconsisten* OR manipulate* OR decept* OR dishonest OR deceive* OR misreport* OR fraud* OR lying OR false)) | 10,152 |
| 6 | 3 OR 4 OR 5 | 434,073 |
| 7 | 1 AND 2 AND 6 | 2,342 |
| 8 | Limit: 1980-01-01 to present | 2,336 |

**Date of Search:** December 13, 2019 (updated July 23, 2021)

**Database:** Emerald Insight

**Platform:** Emerald

**Date of Search:** December 18, 2019 (updated July 26, 2021)

abstract:“financial performance” AND (abstract:“ceo*“) AND (bonus*) = 13

abstract:“firm performance” AND (abstract:“ceo*“) AND (bonus*) = 55

abstract:“corporate earnings” AND (abstract:“ceo*“) AND (“bonus*“) = 1

abstract:“financial performance” AND (abstract:“ceo*“) AND (compensation) = 30

abstract:“firm performance” AND (abstract:“ceo*“) AND (compensation) = 108

abstract:“financial performance” AND (abstract:“ceo*“) AND (salary) = 16

abstract:“firm performance” AND (abstract:“ceo*“) AND (salary) = 58

abstract:“pay for performance” AND (ceo*) = 35

abstract:“performance pay” AND (ceo*) = 5

abstract:“performance based pay” AND (ceo*) = 1

abstract:“executive pay” AND (“ceo*“) = 27

abstract:“fraud” AND (“ceo*“) AND (salary) = 40

abstract:“fraud” AND (“ceo*“) AND (compensation) = 83

abstract:“fraud” AND (“ceo*“) AND (bonus*) = 37

abstract:“fraud” AND (“ceo*“) AND (“pay for performance”) = 3

abstract:“misreport*“ AND (“ceo*“) AND (salary) = 3

abstract:“misreport*“ AND (“ceo*“) AND (compensation) = 7

abstract:“misreport*“ AND (“ceo*“) AND (bonus*) = 5

abstract:“misreport” AND (“ceo*“) AND (“pay for performance”) = 1

abstract:“restatement” AND (“ceo*“) AND (salary) = 4

abstract:“restatement” AND (“ceo*“) AND (compensation) = 27

abstract:“restatement” AND (“ceo*“) AND (bonus*) = 5

abstract:“lying” AND (“ceo*“) AND (“salary”) = 66

abstract:“lying” AND (“ceo*“) AND (“compensation”) = 71

abstract:“lying” AND (“ceo*“) AND (“bonus*“) = 45

abstract:“irregular*“ AND (“ceo*“) AND (“salary”) = 3

abstract:“irregular*“ AND (“ceo*“) AND (“compensation”) = 5

abstract:“irregular*“ AND (“ceo*“) AND (“bonus*“) = 4

**Database: Directory of Open Access Journals**

**Platform:** https://doaj.org/

**Date of Search:** February 6, 2020

Basic Search—Articles Only

ceo* AND “financial performance” = 44

ceo* AND “firm performance” = 50

ceo* AND bonus* = 8

“chief executive officer*“ AND bonus* = 4

“chief executive officer*“ AND “financial performance” = 9

“chief executive officer*“ AND “firm performance” = 6

CEO* AND restatement* = 5

“chief executive officer*“ AND restatement* = 0

CEO* AND irregularit* = 8

“chief executive officer*“ AND irregularit* = 0

CEO* AND “pay for performance” = 1

“chief executive officer*“ AND “pay for performance” = 1

“chief executive officer*“ AND “performance-based pay” = 0

CEO* AND “performance-based pay” = 0

CEO* AND revenue = 17

“chief executive officer*“ AND revenue = 5

“chief executive officer*“ AND earnings = 9

CEO* AND earnings = 35

“financial performance” AND “executive compensation” = 9

### Grey Literature

Note: Numbers indicate the number of results for the search string. In most cases, results were screened in the platform and only relevant results were retrieved for formal screening in Covidence.

**National Bureau of Economic Research (NBER) Working Papers** **(RS)**

https://www.nber.org/papers.html

**NOTE:** limit to last 3 years. 1973-present (embargo last 3 years) in ABI/INFORM

**Date of Search:** February 4, 2020 (Updated August 16, 2021)

ceo “financial performance” = 0

ceo “firm performance” = 1

ceo bonus = 0

“chief executive officer” bonus = 0

“chief executive officer” “financial performance” = 0

“chief executive officer” “firm performance” = 6

ceo restatement = 0

“chief executive officer” restatement = 0

ceo irregularity = 0

“chief executive officer” irregularity = 0

ceo “pay for performance” = 0

“chief executive officer” “pay for performance” = 1

ceo “performance-based pay” = 0

“chief executive officer” “performance-based pay” = 0

ceo revenue = 0

“chief executive officer” revenue = 0

ceo earnings = 0

“chief executive officer” earnings = 0

“financial performance” “executive compensation” = 2

**Bureau of Economic Analysis**

https://www.bea.gov/research/papers

Searching title of Papers

**Date of Search:** February 6, 2020 (Updated August 11, 2021)

ceo = 0

ceos = 0

executive = 0

executives = 0

bonus =

bonuses = 0

compensation = 1

performance = 0

irregularity = 0

irregularities = 0

restatement =

restatements = 0

**Board of Governors of the Federal Reserve System**

https://www.federalreserve.gov/publications.htm

Advanced Search; Working Papers only

**Date of Search:** February 5, 2020 (Updated August 11, 2021)

ceo AND “financial performance” = 3

ceo AND “firm performance” = 13

ceo AND bonus = 8

“chief executive officer” AND bonus = 1

“chief executive officer” AND “financial performance” = 0

“chief executive officer” AND “firm performance” = 0

CEO AND restatement = 2

“chief executive officer” AND restatement = 0

“chief executive officer” AND restatements = 0

CEO AND irregularity = 2

CEO AND irregularities = 2

“chief executive officer” AND irregularities = 0

CEO AND “pay for performance” = 8

“chief executive officer” AND “pay for performance” = 0

“chief executive officer” AND “performance-based pay” = 0

CEO AND “performance-based pay” = 1

CEO AND revenue = 28

“chief executive officer” AND revenue = 3

“chief executive officer” AND earnings = 0

CEO AND earnings = 29

“financial performance” AND “executive compensation” = 2

**Federal Reserve Economic Data (FRED) St. Louis FED**

https://fred.stlouisfed.org/

**Date of Search:** February 10, 2020 (Updated August 11, 2021)

ceo AND “financial performance” = 1

ceo AND “firm performance” = 4

ceo AND bonus = 2

“chief executive officer” AND bonus = 0

“chief executive officer” AND “financial performance” = 0

“chief executive officer” AND “firm performance” = 0

CEO AND restatement = 0

“chief executive officer” AND restatement = 0

CEO AND irregularity = 4

“chief executive officer” AND irregularity = 0

CEO AND “pay for performance” =

“chief executive officer” AND “pay for performance” = 0

“chief executive officer” AND “performance-based pay” = 0

CEO AND “performance-based pay” = 0

CEO AND revenue = 11

“chief executive officer” AND revenue = 1

“chief executive officer” AND earnings = 1

CEO AND earnings = 13

“financial performance” AND “executive compensation” = 0

**AEA Papers & Proceedings**

https://www.aeaweb.org/journals/pandp

Article search, Title & Abstract

2018 forward only (previous years indexed in Business Source Ultimate)

**Date of Search:** February 6, 2020 (Updated August 11, 2021)

ceo = 0

ceos = 0

executive = 0

executives = 0

bonus = 0

bonuses = 0

compensation = 0

performance = 7

irregularity = 0

irregularities = 0

restatement = 0

restatements = 0

**Social Sciences Research Network (SSRN)**

https://www.ssrn.com/index.cfm/en/

Advanced Search

Search Terms(s), Title, Abstract, & Keywords; All Dates;

SSRN Networks to Refine Search:

Economics, Finance, Management, Organization Series, Corporate Governance, Entrepreneurship, Accounting

**Date of Search:** February 23, 2020 (Updated August 30, 2021)

ceo* “financial performance” bonus* = 23

“chief executive officer*“ “financial performance” bonus* = 2

ceo* “financial performance” incentive* = 68

“chief executive officer*“ “financial performance” incentive* = 5

ceo* “firm performance” bonus* = 65

“chief executive officer” “firm performance” bonus* = 3

ceo* “firm performance” incentive* = 155

“chief executive officer” “firm performance” incentive* = 10

ceo* “pay for performance” bonus* = 51

“chief executive officer” “pay for performance” bonus* = 2

ceo* “pay for performance” incentive* = 170

“chief executive officer” “pay for performance” incentive* = 10

ceo* restatement* incentive* = 2

“chief executive officer” restatement* incentive* = 0

ceo* irregularit* incentive* = 0

“chief executive officer” irregularit* incentive* = 0

“financial performance” “executive compensation” = 283

**IDEAS/ Research Papers in Economcs (RePEc)**

https://ideas.repec.org/

**NOTE:** Searched 1980 – 1999 (2000-present covered in ABI/INFORM)

**Date of Search:** February 5, 2020

TI: ceo + “financial performance” = 1

AB: ceo + “financial performance” = 8

TI: ceo + “firm performance” = 9

AB: ceo + “firm performance” = 38

TI: ceo + bonus = 1

AB: ceo + bonus = 17

TI: “chief executive officer” + bonus = 1

AB: “chief executive officer” + bonus = 17

TI: “chief executive officer” + “financial performance” = 1

AB: “chief executive officer” + “financial performance” = 8

TI: “chief executive officer” + “firm performance” = 9

AB: “chief executive officer” + “firm performance” = 38

TI: ceo + restatement = 0

AB: ceo + restatement = 0

TI: “chief executive officer” + restatement = 0

AB: “chief executive officer” + restatement = 0

TI: ceo + irregularit = 0

AB: ceo + irregularit = 0

TI: “chief executive officer” + irregularit = 0

AB: “chief executive officer” + irregularit = 0

TI: ceo + “pay for performance” = 2

AB: ceo + “pay for performance” = 15

TI: “chief executive officer” + “pay for performance” = 2

AB: “chief executive officer” + “pay for performance” = 15

TI: ceo + “performance-based pay” = 0

AB: ceo + “performance-based pay” = 1

TI: “chief executive officer” + “performance-based pay” = 0

AB: “chief executive officer” + performance-based pay” = 1

TI: ceo + revenue = 0

AB: ceo + revenue = 3

TI: “chief executive officer” + revenue = 0

AB: “chief executive officer” + revenue = 3

TI: ceo + earnings = 2

AB: ceo + earnings = 12

TI: “chief executive officer” + earnings = 2

AB: “chief executive officer” + earnings = 10

TI: “financial performance” + “executive compensation” = 2

AB: “financial performance” + executive compensation” = 3

**Centre for Economic Policy Research**

https://cepr.org/

Search performed using Google domain search with screening of all search results for relevant items.

**Date of Search:** February 9, 2020 (Updated August 11, 2021)

ceo* AND “financial performance” AND bonus* site:.cepr.org = 30

ceo* AND “firm performance” AND bonus* site:.cepr.org = 127

ceo* AND “financial performance” AND incentive* site:.cepr.org = 7

ceo* AND “firm performance” AND incentive* site:.cepr.org = 317

ceo* AND restatement* site:.cepr.org = 18

ceo* AND irregularit* site:.cepr.org = 3

ceo* AND “pay for performance” site:.cepr.org = 6

ceo* AND “performance-based pay” site:.cepr.org = 8

“financial performance” AND “executive compensation” site:.cepr.org = 1

“firm performance” AND “executive compensation” site:.cepr.org = 99

**Conference Board- Business Management Research**

https://www.conference-board.org/ea/search.cfm

Search performed using Google domain search

**Date of Search:** February 7, 2020 (Updated August 16, 2021)

ceo* AND “financial performance” AND bonus* site:.conference-board.org = 44 (1)

Ceo* AND “firm performance” AND bonus* site:.conference-board.org = 9 (0)

Ceo* AND “financial performance” AND incentiv* site:.conference-board.org = 60 (0)

Ceo* AND “firm performance” AND incentiv* site:.conference-board.org = 23 (0)

Ceo* AND “pay for performance” AND bonus* site:.conference-board.org = 63 (2)

Ceo* AND “pay for performance” AND incentiv* site:.conference-board.org = 100 (0)

Ceo* AND restatement* AND incentive* site:.conference-board.org = 60 (0)

Ceo* AND irregularit* AND incentive* site:.conference-board.org = 4 (0)

“financial performance” AND “executive compensation” site:.conference-board.org = 86 (0)

**Business Council of Canada**

https://thebusinesscouncil.ca/

SEARCH KEYWORD(S); Publication Types: Articles, Reports; RELATED ISSUES: Corporate / Public Governance; Macroeconomic and Fiscal; North America

Date: From 01/01/1980 To 03/01/2020

**Date of Search:** February 11, 2020 (Updated August 16, 2021)

ceo AND financial performance = 5

ceos AND financial performance = 2

ceo AND firm performance = 4

ceos AND firm performance = 1

ceo AND bonus = 0

ceos AND bonus = 0

ceos AND bonuses = 0

chief executive officer AND bonus = 1

chief executive officers AND bonus = 1

chief executive officers AND bonuses = 1

chief executive officer AND financial performance = 1

chief executive officer AND firm performance = 2

ceo AND restatement = 0

ceos AND restatement = 0

chief executive officer AND restatement = 0

chief executive officer AND restatements = 0

ceo AND irregularity = 0

ceo AND irregularities = 0

chief executive officer AND irregularities = 0

ceo AND pay for performance = 5

chief executive officer AND pay for performance = 2

chief executive officer AND performance-based pay = 0

chief executive officer AND performance based pay = 2

ceo AND performance-based pay = 0

ceo AND performance based pay = 4

ceo AND revenue = 5

chief executive officer AND revenue = 4

chief executive officer AND earnings = 1

ceo AND earnings = 2

financial performance AND executive compensation = 2

### Handsearching

**Advances in Business Research**

Manual screening of titles/abstracts

Date range: All (2010 to present)

Date of Search: 2-9-20

**Academy of Management Review**

Manual screening of TI/ABs

Date Range: 2019-Present, included “In-press” articles

Date of Search: 2-24-20

**Academy of Management Annals**

Manual screening of titles/abstracts

Date range: 2019-Present, included “In-press” articles

Date of search: 2-10-20

**International Conference on Economics, Business and Management (ICEBM) -Journal of Economics, Business and Management**

Scanned TOCs and programs of proceedings

Data range: All available (2011 to 2017)

Date of search: 2/9/2020

**International Conference on Advances in Management Sciences (ICAMS) - Journal of Advanced Management Science**

Manual screening of Tables of Contents

Date range: 2013-2019

Date of search: 02/24/2020

**Academy of International Business (AIB) -Proceedings of the Annual Meeting of the Academy of International Business**

Manual screening of titles/abstracts

Data range: 1998-2012

Date of search: 2/9/2020

### Supplementary Searching

Forward citation searching:

Method: Searched Google Scholar for citing references of all included studies. If zero, checked Web of Science and Scopus. Screened studies for potential relevance and only potentially relevant studies retrieved for formal screening in Covidence.

Number of relevant records: 92

Backward citation searching:

Method: Hand-searched reference of included studies.

Number of relevant references: 35
